# A prospective three-year follow-up study on the clinical significance of anti-neuronal antibodies in acute psychiatric disorders

**DOI:** 10.1038/s41598-019-56934-6

**Published:** 2019-12-31

**Authors:** M. B. Schou, S. G. Sæther, O. K. Drange, E. Brenner, J. Crespi, L. Eikenes, M. S. Mykland, C. Pintzka, A. K. Håberg, T. Sand, A. Vaaler, D. Kondziella

**Affiliations:** 1 Department of Mental Health, Norwegian University of Science and Technology (NTNU), Trondheim, Norway; 2 Division of Mental Health Care, St. Olavs hospital, Trondheim University Hospital, Trondheim, Norway; 3 Department of Neuromedicine and Movement Science, Norwegian University of Science and Technology (NTNU), Trondheim, Norway; 4 Department of Neurology and Clinical Neurophysiology, St. Olavs hospital, Trondheim University Hospital, Trondheim, Norway; 5 Norwegian Advisory Unit on Headaches, Trondheim, Norway; 6 Department of Circulation and Medical Imaging, Norwegian University of Science and Technology (NTNU), Trondheim, Norway; 7 Department of Radiology and Nuclear Medicine, St. Olavs hospital, Trondheim University Hospital, Trondheim, Norway; 8 Department of Neurology, Rigshospitalet, Copenhagen University Hospital, Copenhagen, Denmark; 9 Faculty of Health and Medical Sciences, Copenhagen University, Copenhagen, Denmark

**Keywords:** Autoimmune diseases, Depression, Psychosis

## Abstract

The clinical significance of anti-neuronal antibodies for psychiatric disorders is controversial. We investigated if a positive anti-neuronal antibody status at admission to acute psychiatric inpatient care was associated with a more severe neuropsychiatric phenotype and more frequent abnormalities during clinical work-up three years later. Patients admitted to acute psychiatric inpatient care who tested positive for N-methyl-D-aspartate receptor (NMDAR), contactin-associated protein 2 (CASPR2) and/or glutamic acid decarboxylase 65 (GAD65) antibodies (n = 24) were age – and sex matched with antibody-negative patients (1:2) from the same cohort (n = 48). All patients were invited to follow-up including psychometric testing (e.g. Symptom Checklist-90-Revised), serum and cerebrospinal fluid (CSF) sampling, EEG and 3 T brain MRI. Twelve antibody-positive (ab+) and 26 antibody-negative (ab−) patients consented to follow-up. Ab+ patients had more severe symptoms of depression (p = 0.03), psychoticism (p = 0.04) and agitation (p = 0.001) compared to ab− patients. There were no differences in CSF analysis (n = 6 ab+/12 ab−), EEG (n = 7 ab+/19 ab−) or brain MRI (n = 7 ab+/17 ab−) between the groups. In conclusion, anti-neuronal ab+ status during index admission was associated with more severe symptoms of depression, psychoticism and agitation at three-year follow-up. This supports the hypothesis that anti-neuronal antibodies may be of clinical significance in a subgroup of psychiatric patients.

## Introduction

The discovery that anti-neuronal antibodies cause distinct clinical syndromes with prominent neuropsychiatric symptoms has shown a remarkable link between immunology and psychiatry^1,2^. Several studies have estimated the prevalence of antibodies in patients with primary psychiatric disorders (including antibodies against N-methyl-D-aspartate receptor (NMDAR), contactin-associated protein 2 (CASPR2), and glutamic acid decarboxylase 65 (GAD65))^3–8^. However, if anti-neuronal antibodies are clinically important in psychiatric patients who do not fulfill criteria of autoimmune encephalitis remains unknown.

Evidence from preclinical models suggest that NMDAR antibodies found in psychiatric patients have pathogenic potential^4,9^. It was recently demonstrated that NMDAR antibodies from patients with schizophrenia alter the surface dynamics and organization of NMDARs in these patients, but not in healthy controls^10^. Most researchers examining the phenotype of anti-neuronal antibody-positive psychiatric patients have focused on patients with psychotic disorders and report quite similar phenotypes in patients with and without NMDAR antibodies as measured with the Positive and Negative Syndrome Scale (PANSS)^4,5^. However, there is a lack of studies investigating the clinical significance of anti-neuronal antibodies in psychiatric patients with non-psychotic phenotypes.

We recently identified NMDAR, CASPR2 and/or GAD65 antibodies (Immunoglobulin (Ig) G, IgA and/or IgM isotypes) retrospectively in 11.6% (107 out of 925) of unselected patients admitted to acute psychiatric inpatient care^6^. In a case-control study, we further found that the psychiatric phenotypes during acute admission were similar in patients with and without antibodies^11^.

In the present paper, to further evaluate the clinical significance of anti-neuronal antibodies, we assessed a subgroup of these patients three years after the index admission, using a multimodal approach including psychometric testing, cerebrospinal fluid (CSF) analysis, electroencephalography (EEG) and brain magnetic resonance imaging (MRI). We hypothesized that patients who were anti-neuronal antibody-positive at index admission would have a) more severe neuropsychiatric symptoms and b) a higher frequency of findings in CSF and on EEG and brain MRI suggestive of previous (or ongoing) autoimmune encephalitis compared to antibody-negative patients (Table 1).

**Table 1:** Hypotheses tested and examinations performed at three-year follow-up.

Hypotheses	Assessment tool	Variables
Anti-neuronal antibody-positive patients have more severe neuropsychiatric symptoms.	SCL-90-R	Depression, anxiety, psychoticism, paranoid ideation, global symptom severity.
	ISI	Sleep disturbances.
	PANSS-EC	Agitation.
	ACE-R	Cognitive function.
Anti-neuronal antibody-positive patients have more frequent signs of neuroinflammation and blood brain barrier dysfunction.	Lumbar puncture (CSF)	White blood cells, IgG index, oligoclonal bands and albumin quotient.
Anti-neuronal antibody-positive patients have more pathological EEG findings (focus on temporal lobe).	EEG	Epileptiform and slow wave activity.
	qEEG	Temporal alpha, theta and delta activity (spectral amplitude).
Anti-neuronal antibody-positive patients have more atrophy and microstructural changes of selected cerebral structures (focus on temporal lobe).	Brain MRI	Volume of total cerebral cortex, hippocampus, limbic system and cerebral white matter.
		DTI measures of mean diffusivity and fractional anisotropy of the total white skeleton, cingulum and uncinate fascicle.
		DKI measures of mean kurtosis of the hippocampus and uncinate fascicle.

ACE-R; Addenbrooke’s Cognitive Examination Revised, CSF; cerebrospinal fluid, DKI; diffusion kurtosis imaging, DTI; diffusion tensor imaging, EEG; electroencephalography, Ig; immunoglobulin, ISI; Insomnia Severity Index, MRI; magnetic resonance imaging, PANSS-EC; Positive and Negative Syndrome Scale Excited Component, qEEG; quantitative electroencephalography, SCL-90-R; Symptom Checklist-90 Revised.

## Results

### Patients

**Figure 1:**
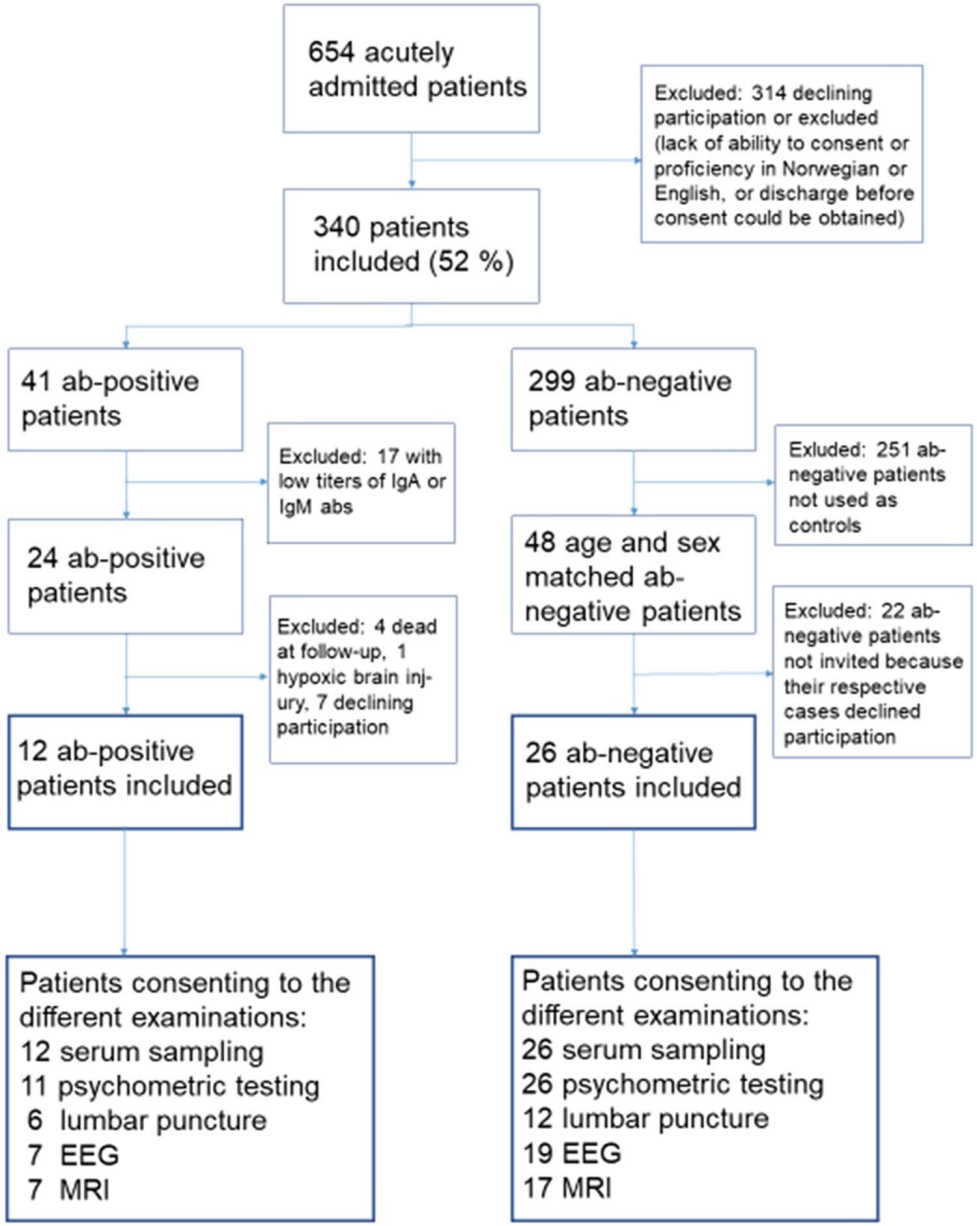
Patient flow and number of patients consenting to the different examinations. Ab; antibody, Abs; Antibodies, EEG; electroencephalography, Ig; immunoglobulin, MRI; magnetic resonance imaging.

A total of 12 of the 24 (50%) antibody-positive patients and 26 of 48 (54%) of the antibody-negative patients participated in the follow-up study, see Fig. 1 for patient flow. Females were over-represented among the included patients (seven out of 12) compared to those not included (one out of 12, p = 0.03), but age was similar between the groups (mean 47.0 years (SD 17.2) versus 50.5 years (SD 13.9), p = 0.59). Supplementary Table 1 shows antibody status and reasons for exclusions for every excluded patient individually.

Antibody-positive and -negative patients participating in the follow-up study did not differ significantly regarding psychiatric diagnosis at index admission (2011–2012), educational level, use of psychopharmacological medication, alcohol or substances, or time to follow-up (Table 2). Supplementary Table 2 shows diagnoses, psychometric test results and pathological CSF, EEG and MRI findings for all antibody-positive patients individually.

**Table 2:** Demographic and clinical characteristics of patients included in the follow-up study.

	Ab-positive patients (n = 12)	Ab-negative patients (n = 26)	p^a^
Age, mean (SD)	47.0 (17.2)	44.5 (13.2)	0.63^b^
Sex, male %	42	50	0.63
Educational level, %			0.81
≤9 years	42	42	
10–12 years	33	42	
>12 years	25	15	
Psychiatric diagnosis at index admission (ICD-10), n (%)			0.80
Psychotic disorders (F20-F29)	2 (17%)	3 (12%)	
Affective disorders (F30-F39)	6 (50%)	12 (46%)	
Other psychiatric diagnoses^c^	4 (33%)	11 (42%)	
Ab status at index admission^d^	5 NMDAR (1 IgG, 3 IgM, 2 IgA), 3 CASPR2 (3 IgG), 6 GAD65 (6 IgG)	—	
Months between index admission and clinical follow-up, mean (SD)	40.6 (3.1)	40.3 (2.1)	0.79^b^
**Psychopharmacological medication at follow-up, n (%)**			
Antipsychotics^e^	6 (50%)	10 (38%)	0.50
Antidepressants^f^	3 (25%)	5 (19%)	0.69
Mood stabilizers^g^	4 (33%)	9 (35%)	0.94
Substance use 6 months prior to follow-up^h^, n (%)	3 (27%)^i^	4 (15%)	0.40
Median number of alcohol units per week the 4 weeks prior to follow-up, median (IQR)	0 (0, 1.5)^i^	0.4 (0, 1.1)	0.46

Ab; antibody, CASPR2; contactin-associated protein 2, GAD65; glutamic acid decarboxylase-65, ICD-10; international classification of diseases-10, Ig; immunoglobulin, IQR; interquartile range, NMDAR; N-methyl-D-aspartate receptor; SD; standard deviation. Significance level 0.05. ^a^chi square test when not otherwise specified. ^b^Student’s t-test. ^c^Mental and behavioural disorders due to psychoactive substance use (F10–19) n = 5, Neurotic, stress–related and somatoform disorders (F40–49) n = 6, Disorders of adult personality and behaviour (F60–69) n = 2 and Without spesific psychiatric diagnosis (Z00-Z99) n = 2. ^d^2 patients positive for both NMDAR and GAD65 antibodies and 1 patient positive for NMDAR IgA and IgM antibodies (See Supplementary Table 2 for full list of antibody status and endpoint titer). ^e^Quetiapine (n = 8), olanzapine (n = 2) paliperidone (n = 1), zuclopenthixol (n = 1), aripiprazole (n = 4), levomepromazine (n = 1), risperidone (n = 1), clozapine (n = 1). ^f^Citalopram (n = 2), escitalopram (n = 2), minaserine (n = 2), clomipramine (n = 1), mirtazapine (n = 1), venlafaxine (n = 1). ^g^Lamotrigine (n = 7), valproate (n = 5), lithium (n = 4). ^h^Benzodiazepines (n = 5), tetrahydrocannabinol (n = 4). ^i^n = 11.

### Neuropsychiatric symptomatology

Antibody-positive patients had significantly more severe symptoms of depression, psychoticism and agitation as compared to antibody-negative patients. Symptom scores of anxiety, paranoid ideation, cognitive dysfunction, and the global severity index (GSI) were also higher among antibody-positive compared to antibody-negative patients, but these findings were not statistically significant (Table 3). The brief neurological screening did not show any significant differences between the antibody-positive and -negative patients (data not shown).

**Table 3:** Neuropsychiatric symptomatology at follow-up.

Symptom variables	Ab-positive patients (n = 11)	Ab-negative patients (n = 26)	p^a^	Effect size^b^
Depression (0–4)^c^, median (IQR)	2.2 (1.3, 3.3)	0.9 (0.5, 1.8)	**0.03**	0.36
mean ± SD	2.1 ± 1.1	1.2 ± 1.0		
Anxiety (0–4)^c^, median (IQR)	1.9 (0.4, 2.2)	0.7 (0.4, 1.3)	0.18	0.22
mean ± SD	1.5 ± 1.1	0.9 ± 0.9		
Psychoticism (0–4)^c^, median (IQR)	0.8 (0.4, 1.3)	0.3 (0.0, 0.8)	**0.04**	0.33
mean ± SD	1.0 ± 0.9	0.5 ± 0.8		
Paranoid ideation (0–4)^c^, median (IQR)	1.7 (0.2, 2.0	0.4 (0.1, 1.6)	0.18	0.22
mean ± SD	1.4 ± 0.9	0.9 ± 1.0		
Global severity index^c^ (0–4), median (IQR)	1.5 (0.7, 2.2)	0.6 (0.4, 1.3)	0.10	0.27
mean ± SD	1.5 ± 1.0	0.9 ± 0.8		
Sleep disturbances (0–28)^d^mean ± SD	10.5 ± 8.4	12.2 ± 6.9	0.54^e^	0.22 ^f^
Agitation (5–35)^g^, median (IQR)	6 (5, 8)	5 (5, 5)	**0.001**	0.59
mean ± SD	6.5 ± 1.6	5.1 ± 0.4		
Cognitive function (0–100)^h^, median (IQR)	88 (79, 92)	92 (83, 94)	0.22	0.20
mean ± SD	83.1 ± 15.4	88.6 ± 8.7		

Ab; antibody, IQR; interquartile range, SD; standard deviation. Significance level 0.05. ^a^Mann-Whitney U test when not otherwise specified. ^b^r when not otherwise specified. ^c^Symptom Checklist-90 Revised. ^d^Insomnia Severity Index. ^e^Student’ t test. ^f^Cohen’s d. ^g^Positive and Negative Syndrome Scale Excited Component. ^h^Addenbrooke’s Cognitive Examination Revised.

### Serum and CSF analyses

Two out of 12 antibody-positive patients (17%) were still serum antibody-positive at follow-up (Table 4). Both had lower titers of NMDAR antibodies compared to at index admission. One patient had a reduction of IgA antibody titer from 1:1000 to 1:320 and IgM antibody titer from 1:1000 to 1:10, and the other a reduction of IgM antibody titer from 1:100 to 1:32. The latter patient also had a slightly increased CSF/serum albumin quotient. We did not observe pathological CSF findings in any other patient at follow-up (Table 3).

**Table 4:** Serum and CSF findings at follow-up.

Serum/CSF findings	Ab-positive patients (n = 6)	Ab-negative patients (n = 12)	p^a^	Effect size (ɸ)
	Pathological (yes/no), n (%)	Pathological (yes/no), n (%)		
Serum anti-neuronal abs^b^	2 (17)	0 (0)	0.094	0.35
CSF anti-neuronal abs	0 (0)	0 (0)	1	n/a
CSF pleocytosis (≤5 cells)	0 (0)	0 (0)	1	n/a
Ig G index (<0.70)	0 (0)	0 (0)	1	n/a
CSF oligoclonal bands	0 (0)	0 (0)	1	n/a
CSF/serum albumin quotient^c^	1 (17)	0 (0)	0.33	0.34

Ab; antibody, abs; antibodies, CSF; cerebrospinal fluid, Ig; immunoglobulin, n/a; not applicable.

Significance level 0.05. ^a^Fisher’s exact test. ^b^Antibody-positive patients, n = 12, antibody-negative patients, n = 26. ^c^Normal value is age related ((4 + age/15) * 10^−3^)^52^.

### EEG/qEEG

Two antibody-positive and two antibody-negative patients had focal or generalized slow wave activity. One antibody-negative patient had epileptiform activity (Table 5). qEEG measurements of temporal alpha, theta or delta amplitude did not differ between antibody-positive and -negative patients. Asymmetry was not observed as the side- and side × group factors were non-significant (Supplementary Table 3). There were no differences in epochs analyzed for qEEG variables between antibody-positive and -negative patients (mean 3.3 (SD 0.8) and 3.8 (SD 0.9) minutes, respectively, p = 0.30).

**Table 5:** EEG findings at follow-up.

*EEG findings*	Ab-positive patients (n = 7)	Ab-negative patients (n = 19)	p^a^	Effect size (ɸ)
	Pathological, (yes/no), n (%)	Pathological, (yes/no), n (%)		
Epileptiform activity	0	1^b^	1	0.12
Focal or generalized slow activity	2^c^	2^d^	0.29	0.22
Normal EEG	5	16	0.59	0.14

Ab; antibody, EEG; electroencephalography. Significance level 0.05. ^a^Fisher’s exact test. ^b^Intermittent focal epileptiform. ^c^Generalized slowing (n = 1), intermittent generalized slowing (n = 1). ^d^Intermittent bilateral frontotemporal slowing (n = 1), intermittent focal slowing (n = 1).

### Brain MRI

There were no significant differences in volume of cortical gray matter, hippocampus, limbic system or cerebral white matter volume between antibody-positive and -negative patients. The diffusion tensor imaging (DTI) and diffusion kurtosis imaging (DKI) analyses revealed not significant differences of mean fractional anisotropy (FA) or mean diffusity (MD) on the total white matter skeleton, or FA, MD or mean kurtosis (MK) in the ROI analyses, between the two groups (Supplementary Table 4). Clinical brain MRI did not reveal any findings that were considered relevant for psychiatric symptomatology or indicative of previous or ongoing encephalitis (Supplementary Table 5).

#### Post hoc analyses

Post hoc analyses for IgG antibody-positive cases (n = 9) were performed (excluding IgA and IgM antibody-positive cases). The results of these comparisons were similar to the main analyses for all variables except slightly lower p-values for paranoid ideation (p = 0.09) and the global severity index (p = 0.04).

In qEEG post hoc analyses the alpha, theta and delta amplitude did not differ significantly between antibody-positive and -negative patients for any brain region (only the temporal lobe was included in the main analyses) (Supplementary Table 6).

## Discussion

Three years after admission to acute psychiatric inpatient care, patients who were serum-positive to anti-neuronal antibodies during index admission had significantly more severe symptoms of depression, psychoticism and agitation compared to the serum-negative patients. However, in the multimodal work-up including neurological examination, CSF analysis, EEG and brain MRI we found that the increased symptom burden was not accompanied by findings suggestive of previous or on-going autoimmune encephalitis. To our knowledge this is the first long-term follow-up study of anti-neuronal antibody-positive psychiatric patients.

### Neuropsychiatric symptomatology

An antibody-positive status during index admission predicted significant more severe symptoms of depression and psychoticism, as measured by the Symptom Checklist-90-Revised (SCL-90-R), at three-year follow-up. In order to evaluate the clinical significance of these symptomatic differences, we compared our findings to a previous study examining the SCL-90-R-scores of an asymptomatic, a moderately symptomatic and a severely symptomatic population^12^. The symptoms of depression and psychoticism in our antibody-positive subgroup corresponded to the severely symptomatic population in the cited study, whereas the antibody-negative patients had symptom scores similar to the moderately symptomatic population. Further, the effect size of the symptom differences between antibody-positive and -negative patients were medium to large indicating clinically meaningful differences in the symptom burden between the two groups.

The degree of agitation as measured by the Positive and Negative Syndrome Scale Excited Component (PANSS-EC) was significantly higher in antibody-positive patients compared to antibody-negative patients. A large proportion of the antibody-positive patients (6 out of 11) had PANSS-EC-scores > 5, whereas the corresponding number was one out of 26 among antibody-negative patients. Despite the highly statistically significant difference between the groups, it may be difficult to use this finding in the clinical setting, due to the low scores in both groups. However, even a small increase in agitation could be of relevance for the patients in daily life. We recommend that future studies perform a more thorough assessment of the components of agitation, which may reveal clinically important phenotypic differences between psychiatric patients with and without anti-neuronal antibodies.

Antibody-positive patients had more severe symptom scores, although not statistically significant, on all variables assessed except sleep disturbances. These findings support that patients who are serum-positive to anti-neuronal antibodies during index admission have a more severe symptom burden at three-year follow-up compared to antibody-negative patients.

In a post hoc analysis excluding IgA and IgM antibody-positive patients from the case group, the difference in total symptom burden (GSI) between the two groups reached statistical significance. This may indicate that the association between symptom severity and antibody-positive status is stronger for IgG positive patients, which fits well with the fact that all known pathogenic antibodies in autoimmune encephalitides are of the IgG isotype^13^.

To our knowledge, there is only one previous longitudinal study of anti-neuronal antibody-positive psychiatric patients. In a sample of first-episode psychosis patients, the authors reported similar functioning and number of contacts with mental health services in antibody-positive and -negative patients at 6-month follow-up^5^. In this study, the severity of neuropsychiatric symptoms was not assessed at follow-up.

### Serum and CSF findings

Only 2 out of 12 antibody-positive patients were still serum-positive at follow-up. One of these also had elevated CSF/serum albumin quotient, indicating blood brain barrier dysfunction, but both had lower titers of antibodies compared to at index admission. Few studies have examined the presence of anti-neuronal antibodies in psychiatric patients longitudinally. The authors of one study reported that the level of antibodies targeting the NMDAR NR2 subunit increased during acute mania and normalized in the euthymic phase^14^. Our findings also indicate that the titers of anti-neuronal antibodies in patients with psychiatric disorders differ between acute and more stable phases. Future studies need to address the possible time association between antibody titers and symptom severity.

Of note, none of our antibody-positive or -negative patients had anti-neuronal antibodies or signs of neuroinflammation in the CSF at follow-up. Authors of previous studies have reported a very low prevalence (0–3%) of anti-neuronal antibodies in CSF of psychiatric patients^15–18^. On the other hand, others have questioned the reliability for CSF antibody detection in patients with low titers of anti-neuronal antibodies (i.e. psychiatric patients)^19^. These authors proposed that the brain acts as an “immunoprecipitator”; that is, anti-neuronal antibodies in low titers are prevented from drainage into the CSF because of their binding to the neuronal target. More general signs of neuroinflammation or blood brain barrier dysfunction have been found in a substantial subgroup (15–30%) of patients with psychosis or affective disorder (including increased white blood cell count, IgG index, albumin quotient and/or oligoclonal bands)^15–18^. The patients included in our study were not examined with a lumbar puncture at the index admission. So, to asses a blood brain barrier dysfunction or intrathecal antibody production in the acute phase was not possible in this study. Hence, prospective, longitudinal, controlled CSF studies in patients with severe psychiatric disorders are needed to further investigate the clinical significance of such findings in subgroups of patients^20^.

### EEG/qEEG

Focal or generalized slowing and epileptic activity are the most common pathological EEG findings in patients with autoimmune encephalitis^21^. Although 19% (5 of 26) of the participants in the present study had such findings, there were no significant differences between antibody-positive and -negative patients. This was also true for the quantitative EEG measures of temporal alpha, theta or delta waves. Some studies indicate that pathological EEG findings are more common in anti-neuronal antibody-positive patients during the acute phase of psychotic disorders^17,22^ although this question remains open. Future studies should examine anti-neuronal antibody-positive patients with EEG/qEEG both in the acute and stable phase of psychiatric disorders.

### Brain MRI

There were no morphometric, diffusion or clinical differences on brain MRI between antibody-positive and -negative patients at follow-up. In contrast, morphometric and diffusional characteristics in patients with NMDAR encephalitis show more often hippocampal atrophy^23^ or extensive white matter changes^24^ at follow-up. Clinical MRI findings in follow-up of patients with NMDAR encephalitis^24,25^ or CASPR2 encephalitis^25^ have revealed both normal and unspecific findings, whereas GAD65 encephalitis patients more often develop hippocampal sclerosis^25^.

Brain MRI studies in psychiatric patients with anti-neuronal antibodies are rare. A published abstract on patients at risk of psychosis revealed an interaction between blood brain barrier permeability status, as measured by S100B levels, and larger hippocampal volumes in anti-neuronal antibody-positive patients^26^. This association was not found for antibody-negative patients indicating the possibility that the clinical relevance of anti-neuronal antibodies in psychiatric patients depend on blood brain barrier permeability. However, the data presented in the abstract are limited and caution should be taken interpreting the results. With the effect sizes we found for hippocampal volume differences, one would need approximately 70 cases and 140 controls to ascertain significant group differences (power calculation was performed for left hippocampal volume). It follows that large multi-center studies would be needed to investigate this issue further.

### Limitations

The main limitations in the present study are the low sample size and heterogeneity of antibody findings in the case group. Several statistical comparisons were made in the study, which makes it prone to type I statistical errors. However, the symptom differences found have a moderate to high effect size, which lowers the risk of false positive findings. We chose a significance level of 0.05 since a strict correction for multiple testing would result in type II error inflation^27^. It should be emphasized that the study design does not allow conclusions on causality between anti-neuronal antibody status and symptom severity. Even though the groups are matched on age and sex, it is possible that other confounding factors account for the association. The study also holds a high risk of selection bias. Patients with the most severe psychiatric phenotypes more often decline participation or lack capability to consent to participate in studies. It is thus difficult to extrapolate findings to patients with the most severe psychiatric phenotypes. Lastly, the analytical method used for antibody detection, a fixed cell-based assay, has shown a good sensitivity and specificity for detecting NMDAR antibodies in patients with autoimmune encephalitis^28^. However, one study suggest that live cell-based assays have a higher sensitivity in patients with psychiatric disorders^29^. A consensus on the gold standard for detecting anti-neuronal antibodies in psychiatric patients (see Jezequel *et al*. for a discussion on antibody detection methods^29^) is needed.

## Conclusion

Anti-neuronal antibody-positive status during admission to acute psychiatric inpatient care is associated with a higher degree of depression, psychoticism and agitation at three-year follow-up. This phenotypic difference may indicate that anti-neuronal antibodies are of clinical significance in psychiatric patients, despite the absence of overt autoimmune encephalitis in the present or past. Longitudinal studies with sample sizes large enough to allow conclusions about each individual anti-neuronal antibody are needed to elaborate this issue further.

## Methods

### Setting and patients

This was a prospective three-year follow-up study of patients admitted to acute psychiatric inpatient care (St. Olavs hospital, Trondheim University Hospital, Trondheim, Norway) during September 2011 – March 2012. Psychiatric diagnoses at the index admission in 2011–2012 were set according to the International Classification of Diseases (ICD)-10 criteria for research^30^ in a consensus meeting with the physicians and psychologists in charge of the treatment of the patient.

The patient flow of the present study is presented in Fig. 1. A total of 41 out of 340 patients tested positive to one or more anti-neuronal antibodies (21 NMDAR, 14 CASPR2 and 9 GAD65, (IgA, IgG or IgM)) during index admission^6^. Of these patients, those with the highest likelihood of having anti-neuronal antibody related disorders were selected based on the following criteria: 1) IgG positivity or 2) high titer of IgA/IgM (1:100 or above). The IgG isotype was selected because of its proven pathogenicity in autoimmune encephalitis^13^. Patients with high titers of the IgA/IgM antibodies were also included because of studies indicating that these isotypes may have clinical relevance when present in higher concentrations^9,31,32^.

This “high probability group” (24 patients) was invited to follow-up together with 48 anti-neuronal antibody-negative patients from the same cohort. Antibody-negative patients were selected randomly among patients with the same sex and age as each antibody-positive patient (age difference between the included antibody-positive and -negative patients were small (median 2.5 years (interquartile range 1.0, 4.0))). Follow-up included evaluation of neuropsychiatric symptomatology, neurological examination, serum and CSF analysis, EEG/qEEG and brain MRI. All patients and examiners (physicians, laboratory personnel, electroencephalographers, neuroradiologists etc.) were blinded for antibody status.

### Examinations at three-year follow-up

#### Demographic characteristics and neuropsychiatric symptomatology

Demographic data and the use of medication, alcohol and substances were assessed by a structured questionnaire at inclusion in the follow-up study. Table 1 summarizes the parameters included in the study. We assessed several neuropsychiatric symptoms frequently seen in encephalitic syndromes associated with NMDAR, CASPR2 and GAD65 antibodies^11^. The degree of depression, anxiety, psychoticism, paranoid ideation and the total symptom burden were evaluated using symptom dimensions and the global severity index (GSI) of the SCL-90-R^33^. The SCL-90-R is a self-report checklist assessing the presence of a broad range of psychiatric symptoms during the last 7 days. The 90 questions are scored from 0 (not at all) to 4 (extreme). Sleep disturbances were evaluated with the self-report questionnaire Insomnia Severity Index (ISI)^34^ (range 0–28, high score indicating high severity of insomnia). Agitation were evaluated using the PANSS-EC^35^, which consists of five items rated from 1–7 (range 5–35, high score indicating high severity of agitation). Cognitive function was assessed by Addenbrooke’s Cognitive Examination Revised (ACE-R)^36^ (range 0–100, lower score indicating lower cognitive function). In addition, we performed neurological examination focusing on oculomotor, vestibular, motor, proprioceptive and cerebellar functions (tandem walking, Romberg’s test, finger-nose-finger test, visual pursuit test, and jump ten times on one foot).

#### Serum and CSF analyses

Serum was collected on the day of the clinical examination and on the day of lumbar puncture. Routine serum and CSF analyses were performed at the hospital laboratory (St. Olav’s Hospital, Trondheim University Hospital). Serum and CSF were frozen at minus 80 °C for anti-neuronal antibody analyses at Euroimmun, Lübeck, Germany. These analyses were performed using biochip mosaics of transfected HEK293 cells expressing the respective recombinant target antigens (NMDAR NR1a subunit, CASPR2 and GAD65)^37^. Samples were classified as positive or negative based on fluorescence intensity of the transfected cells in direct comparison with non-transfected cells and control samples. Endpoint titers refer to the last dilution showing a measurable degree of fluorescence, with 1:10 being the cut-off for positivity in serum and 1:1 in CSF^37^.

#### EEG/qEEG

A 20 minutes clinical routine EEG was recorded in order to examine for epileptiform activity and focal or generalized slow activity. It was recorded digitally including hyperventilation for three minutes and photic stimulation, using a full EEG montage (21 channels), Viasys Nic One 5.11 with a V32 amplifier (Natus Medical Inc, Pleasanton, CA 94566, USA) including quantitative analysis (qEEG). To avoid drowsiness, the subjects were asked to open their eyes every minute as well as being talked to by the technician if drowsiness occurred during the first five minutes of the EEG-recording. A board-certified electroencephalographer evaluated all EEGs.

Average reference montage, band pass filters of 0.5 Hz–70 Hz, and 50 Hz notch filter were used. The EEG-files were scrutinized visually and artefact-free segments were marked for inclusion in Fast Fourier Transform (FFT)-analysis. All epochs with eye blinks, muscular artefacts and electrode artefacts were omitted from the analysis. FFT was performed on artefact-free 2.05-second epochs (cosine tapering, 50% overlap) yielding a power spectrum with 0.49 Hz resolution using Harmonie software (Stellate systems, Quebec, Canada). The time/amplitude series had a sampling frequency of 500 Hz. Amplitude (square root of band power) values (µV) were calculated by summing amplitude across all bins in the 0.49–3.42 Hz (delta), 3.91–7.81 Hz (theta), and 8.3–12.7 Hz (alpha) frequency spectrum. The left and right temporal regions were pre-selected for qEEG due to the frequent involvement of the temporal lobe in encephalopathies associated with anti-neuronal antibodies^23,24,38^. The temporal electrodes T3, T4. T5 and T6 were selected for analysis to reduce the number of variables.

#### Brain MRI

All patients underwent 3 Tesla brain MRI on the same Simens Biograph_mMR scanner with an 8 channel head coil using the same protocol which included T1-weighted 3D scan (e.g. for structural changes and morphometry), T2-weighted sequences and FLAIR (e.g. for edema, strokes, white matter hyperintensities), diffusion-weighted imaging (for cytotoxic edema and other diffusion changes), susceptibility weighted scan (e.g. vasculature and microhemorrhages), diffusion tensor imaging for assessing white matter microstructural integrity) and diffusion kurtosis imaging for evaluating diffusion heterogeneity which might be marker of inflammation^39,40^. For scan details see Supplementary Table 7. The temporal regions were the main focus of brain MRI examinations due to the frequent involvement of the temporal lobe in encephalopaties associated with anti-neuronal antibodies^23,24,38^.

##### Brain morphometry

Quantitative MRI analysis was performed using FreeSurfer 5.3.0, which is documented and freely available online (http://surfer.nmr.mgh.harvard.edu/). All raw T1-weighted images were first converted into NIFTI format and then processed with the fully automated “recon–all” pipeline using the default set of parameters. This includes both a surface-based stream, which is used to segment area and thickness of cortical anatomy and a volume-based stream, which is used to segment and label subcortical tissue classes. The technical details have been thoroughly describes previously^41–44^. Subsequently, the accuracy of the segmentation was visually inspected in each subject. All 24 participants passed the segmentation analysis without the need for removal. Left and right cortical, white matter and hippocampal volumes were extracted, in addition to total estimated intracranial volume. Volumes of the parahippocampal and cingulate cortices were calculated using the available area and thickness measures. Limbic area was defined as the sum of the volume of the amygdala, parahippocampal and cingulate cortex.

##### Brain diffusion characteristics

The DTI and DKI data were used to assess white and grey matter microstructure. The fMRIB Software Library (FSL: http://www.fmrib.ox.ac.uk/fsl) and Diffusion Kurtosis Estimator (DKE: http://academicdepartments.musc.edu/cbi/dki/dke.html) were used for image analysis. Non-brain tissue was removed with the Brain Extraction Tool (BET, FSL). Artifacts due to eddy currents and movements were corrected with eddy, which simultaneously models the effects of diffusion eddy currents and movements on the image. Correction of the susceptibility-induced off-resonance field artefacts were done by topup, a tool for estimating and correcting susceptibility induced distortions^45^ by acquisition of two additional b0 images with opposite phase encoding polarity^45^. From these pairs of images with distortions going in opposite directions, the susceptibility-induced off-resonance field was estimated using a method similar to that described in^45,46^ as implemented in FSL^47^, and the two images were combined into a single corrected one.

DKI and DTI model fitting was performed using DKE and parametric maps were calculated for FA, MD and MK^48^.

Using Tract Based Spatial Statistics (TBSS, FSL)^49^, the mean DTI parameters MD and FA were calculated for the total white skeleton and compared between the antibody-positive and –negative groups.

**Figure 2:**
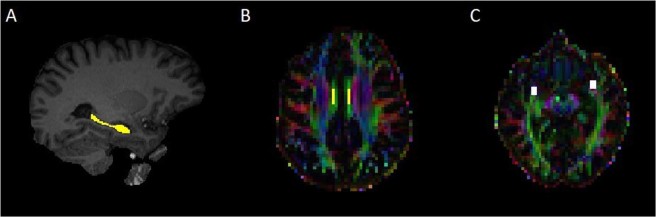
Examples of the delineated a priori bilateral ROIs in the hippocampus (**A**), middle-superior part of cingulum bundle (**B**) and uncinated fasciculus (**C**) in an antibody-positive case.

In addition to the mean FA and MD DTI analysis of the whole white matter skeleton, a ROI analysis was performed in a priori selected bilateral ROIs in white matter in the cingulum and uncinate fascicle and grey matter ROI in hippocampus (Fig. 2). See Supplementary file methods for detailed description of ROI delineation.

DTI parameters FA and MD were extracted for the cingulum, uncinate fascicle and hippocampus ROIs, while the DKI parameter MK were only extracted from the hippocampus and uncinate fascicle ROIs because the DKI volume did not include cingulum. For all the ROIs, mean values were calculated for the DTI and DKI parameters.

### Statistical analyses

#### Demographic data, neuropsychiatric symptomatology, serum and CSF

Categorical data were analyzed using chi square test or Fisher’s exact test. Normality was evaluated by Shapiro-Wilk test and inspection of histogram. Continuous variables were analyzed using Student’s t-test or Mann-Whitney U test.

#### EEG/qEEG

Categorical data were analyzed using Fisher’s exact test. Continuous variables were analyzed using Student’s t-test. Three repeated measures ANOVA was performed on ln-transformed qEEG-amplitudes (alpha, theta and delta bands), with side as within-subject and group as between-subject factors. Mean values, mean + SD and mean –SD was retransformed to the µV-scale for tabulation.

#### Brain MRI

Segmented brain volumes, DTI and DKI data were analyzed using an ANCOVA (age, sex and for segmented brain volumes estimated intracranial volume were used as covariates). Some of these variables were transformed to meet the assumptions of the ANCOVA. The MD measure of cingulum left hemisphere was transformed with square root and the MD measure of cingulum right hemisphere was transformed using log 10. The volume variable of the right limbic system was not possible to transform to meet the criteria homogeneity of variances (the result is reported even if this assumption was violated).

#### Effect sizes

Effect sizes were calculated for; Fisher’s exact test, phi (ɸ) = √(χ^²^/N) (a value of 0.1 is considered a small effect, 0.3 a medium effect, and 0.5 a large effect)^50^; Student’s t-test, d = (M_2_-M_1_)/SD_pooled_, where SD_pooled_ = √((SD_1_^2^ + SD_2_^2^)/2) (a value of 0.2 is considered a small effect, 0.5 a medium effect, and 0.8 a large effect)^50^; Mann-Whitney U test r = z/√N (a value of 0.1 is considered a small effect, 0.3 a medium effect, and 0.5 a large effect)^50^; ANOVA and ANCOVA analysis partial eta squared (η_p_^2^) calculated by SPSS (a value of 0.01 is considered a small effect, 0.09 a medium effect, and 0.25 a large effect).

#### Missing data and double registrations

Missing data on the self-report questionnaire SCL-90-R (24 out of 3330) and ISI (6 out of 259) were imputed using the expectation-maximation (EM) algorithm^51^. Since the SCL-90-R data were positively skewed, data were transformed with the natural logarithm before EM was performed. The missing values were then retransformed before statistical analysis. In the case of double registrations (SCL-90-R: 13, ISI: 1) the mean was used.

#### Post hoc analyses

All variables were re-analyzed including exclusively IgG antibody-positive patients in the case group (IgA and IgM positive patients were included in the main analyses).

In a qEEG post hoc analysis, we analyzed the alpha, theta and delta amplitude for all brain regions (only the temporal lobe was included in the main analyses).

#### Significance level

We set the significance level at p = 0.05 for all comparisons. No formal adjustment for multiple testing was performed. IBM SPSS version 24 was used for statistical analyses.

### Ethics

During their participation in a previous study (Agitation in the Acute Psychiatric Department (clinical trials number NCT01415323)), all patients gave written, informed consent to be contacted for follow-up studies. A new written, informed consent was obtained for the present study. Participants were allowed to restrict themselves from any number of examinations at follow-up. The study was conducted in accordance with the Declaration of Helsinki and approved by The Regional Committee for Medical Research Ethics, Central Norway, REK number 2014/2032.

## Supplementary information

Supplementary Information.

## Data Availability

The datasets generated during and/or analyzed during the current study are available from the corresponding author on reasonable request.
