# Supplementary material for: A prospective three-year follow-up study on the clinical significance of anti-neuronal antibodies in acute psychiatric disorders
Source: Sci Rep. 2019 Dec 31;10:35. doi: 10.1038/s41598-019-56934-6 (PMC6940359; doi:10.1038/s41598-019-56934-6)
Supplement: Supplementary file 1 — Supplementary Information. [file 41598_2019_56934_MOESM1_ESM.docx]

**A prospective three-year follow-up study on the clinical significance of**

**anti-neuronal antibodies in acute psychiatric disorders**

Schou MB^a^, Sæther SG^a^, Drange OK, Brenner E, Crespi J, Eikenes L, Mykland MS,

Pintzka C, Håberg AK, Sand T, Vaaler A, Kondziella D

**Supplementary files**

| Ab-positive patients not included | NMDAR (Ig isotype and titer) | CASPR2 (Ig isotype and titer) | GAD65 (Ig isotype and titer) | Reason for exclusion |
| --- | --- | --- | --- | --- |
| 1 | G, 1:100 |  | G, 1:10 | Dead, myocardial infarction |
| 2 | G, 1:32 |  |  | Dead, hypothermia during alcohol intoxication |
| 3 | M, 1:100 |  |  | Dead, suicide |
| 4 | M, 1:100, A, 1:10 |  |  | Declined |
| 5 | M, 1:100 |  |  | Declined |
| 6 | M, 1:10 |  |  | Declined |
| 7 |  | G, 1:32 |  | Brain injured due to other cause than encephalitis |
| 8 |  | G, 1:10 |  | Declined |
| 9 |  | G, 1:10 |  | Declined |
| 10 |  | A, 1:100 |  | Dead, cancer (unknown type). |
| 11 |  |  | G, 1:10 | Declined |
| 12 |  |  | A, 1:100 | Declined |

**Supplementary table 1.** Anti-neuronal antibody status, endpoint titer and reason of exclusion.

Ab; antibody, CASPR2; contactin-associated protein 2, GAD65; glutamic acid decarboxylase-65, Ig; immunoglobulin

NMDAR; N-methyl D-aspartate receptor.

| **Ab-positive patients included** | **Diagnosis at index admission** | **Antibody status during**  **index admission,**  **(Ig isotype and titer)** | **Global severity index (SCL-90-R)**  **0-4** | **Agitation (PANSS-EC)**  **5-35** | **Insomnia**  **(ISI)**  **0-28** | **Cognitive function (ACE-R)**  **0-100** | **Serum antibody status at follow-up (Ig isotype and titer)** | **CSF/EEG/MRI findings at follow-up** |
| --- | --- | --- | --- | --- | --- | --- | --- | --- |
| 1 | Bipolar disorder, mixed episode | NMDAR (G, 1:10) | 0.70 | 5 | 12 | 92 | ND | Normal/Normal/  Normal |
| 2 | Depressive episode | NMDAR (M, 1:1000 and A, 1:1000) | 0.11 | 6 | 3 | 79 | NMDAR (M, 1:10 and A, 1:320) | NA/NA/NA |
| 3 | Bipolar disorder, manic episode | NMDAR (M, 1:320) and GAD65 (G, 1:10) | 1.81 | 9 | 1 | 42 | ND | Normal/Intermittent generalized slowing/Normal |
| 4 | Adjustment disorder | NMDAR (M, 1:100) | 1.47 | 5 | 19 | 94 | NMDAR (M, 1:32) | Increased albumin quotient/Normal/  Normal |
| 5 | Schizophrenia, paranoid type | NMDAR (A, 1:10) and GAD65 (G, 1:10) | 3.10 | 8 | 20 | 73 | ND | NA/NA/NA |
| 6 | Unspecified mood disorder | CASPR2 (G, 1:100) | 1.97 | 7 | 10 | 90 | ND | Normal/NA/NA |
| 7 | Adjustment disorder | CASPR2 (G, 1:32) | 2.38 | 5 | 23 | 88 | ND | Normal/Normal/  Normal |
| 8 | Schizoaffective disorder, bipolar type | CASPR2 (G, 1:10) | 0.18 | 9 | 2 | 98 | ND | NA/generalized slowing/Normal |
| 9 | Adjustment disorder | GAD65 (G, 1:320) | 2.20 | 7 | 19 | 90 | ND | NA/Normal/Normal |
| 10 | Alcohol induced psychotic disorder | GAD65 (G, 1:100) | 1.53 | 5 | 3 | 81 | ND | Normal/Normal/  Normal |
| 11 | Bipolar disorder, manic episode | GAD65 (G, 1:100) | NA | NA | NA | NA | ND | NA/NA/NA |
| 12 | Depressive episode | GAD65 (G, 1:10) | 0.70 | 5 | 4 | 87 | ND | NA/NA/NA |

**Supplementary table 2**. Antibody-positive patients, diagnosis, antibody status, clinical and paraclinical findings at follow-up

Ab; antibody, ACE-R; addenbrooke’s cognitive examination revised, CASPR2; contactin-associated protein 2, CSF; cerebrospinal fluid, EEG; electroencephalography, GAD65; glutamic acid decarboxylase-65, Ig; immunoglobulin, ISI; insomnia severity index, MRI; magnetic resonance imaging, NA; Not assessed, ND; Not detected, NMDAR; N-methyl D-aspartate receptor, PANSS-EC; positive and negative syndrome scale – excited component, SCL-90-R; symptom checklist-90-revised.

|  | Ab-positive patients (n=7) | | Ab-negative patients (n=19) | | Statistics | | | | | | Effect size |
| --- | --- | --- | --- | --- | --- | --- | --- | --- | --- | --- | --- |
|  |  |  |  |  | Between subjects  *group* effect | | *Side×group* interaction effect | | Within-subjects *side* effect | | (η_p_^2^) between subjects  *group* |
|  | Mean | Mean±SD^a^ | Mean | Mean±SD^a^ | F | p | F | p | F | p |  |
| Alpha, µV |  |  |  |  | 0.37 | 0.55 | 0.035 | 0.85 | 3.64 | 0.07 | 0.015 |
| Left | 5.54 | 3.35, 9.16 | 6.27 | 3.94, 9.95 |  |  |  |  |  |  |  |
| Right | 5.80 | 3.60, 9.34 | 6.63 | 4.02, 10.92 |  |  |  |  |  |  |  |
| Theta, µV |  |  |  |  | 0.58 | 0.45 | 0.19 | 0.67 | 0.15 | 0.70 | 0.024 |
| Left | 4.16 | 2.89, 5.99 | 4.83 | 3.23, 7.21 |  |  |  |  |  |  |  |
| Right | 4.26 | 2.87, 6.33 | 4.82 | 3.12, 7.45 |  |  |  |  |  |  |  |
| Delta, µV |  |  |  |  | 0.15 | 0.70 | 0.0 | 0.99 | 1.96 | 0.18 | 0.006 |
| Left | 5.94 | 4.23, 8.34 | 5.65 | 4.23, 7.53 |  |  |  |  |  |  |  |
| Right | 5.78 | 4.25, 7.86 | 5.50 | 4.08, 7.41 |  |  |  |  |  |  |  |

**Supplementary table 3.** Quantitative EEG findings in the temporal lobes at follow-up.

Left T3,T5 electrode average, Right T4,T6 electrode average. Ab; antibody, EEG; electroencephalography, µV; mikrovolt, SD; standard deviation.

Significance level 0.05. Degrees of freedom for F values (df)= (1,24), ^a^Retransformed mean-SD and mean+SD from log (uV) scale to uV scale

| \| Brain structure \|  \| Ab-positive patients (n=7) \| \| Ab-negative patients (n=17) \| \| p \| Effect size (η_p_^2^) \| \| --- \| --- \| --- \| --- \| --- \| --- \| --- \| --- \| \| **Volume** \|  \| Mean volume^a^, ml (SEM) \| 95 % CI \| Mean volume^a^, ml (SEM) \| 95 % CI \| \| Cortex l.h. \|  \| 220.73 (6.44) \| (207.21-234.25) \| 219.02 (2.64) \| (213.47-224.56) \| 0.81 \| 0.002 \| \| Cortex r.h. \|  \| 222.14 (6.72) \| (208.02-236.26) \| 220.68 (2.76) \| (214.88-226.47) \| 0.84 \| 0.002 \| \| Hippocampus l.h. \|  \| 4.41 (0.25) \| (3.88-4.94) \| 4.13 (0.10) \| (3.91-4.35) \| 0.32 \| 0.054 \| \| Hippocampus r.h. \|  \| 4.57 (0.22) \| (4.11-5.04) \| 4.28 (0.091) \| (4.09-4.47) \| 0.24 \| 0.076 \| \| Limbic System l.h.^b^ \|  \| 12.97 (0.59) \| (11.73-14.20) \| 12.84 (0.24) \| (12.33-13.34) \| 0.84 \| 0.002 \| \| Limbic System r.h.^b^ \|  \| 12.62 (0.52) \| (11.53-13.72) \| 12.24 (0.21) \| (11.79-12.69) \| 0.50 \| 0.025 \| \| White matter l.h. \|  \| 220.85 (9.65) \| 200.58-241.12) \| 229.43 (3.96) \| (221.12-237.75) \| 0.42 \| 0.036 \| \| White matter r.h. \|  \| 222.73 (9.54) \| (202.69-242.78) \| 230.11 (3.91) \| (221.88-238.33) \| 0.48 \| 0.028 \| \| **DTI** \|  \| Mean^c^ \| 95 % CI \| Mean^c^ \| 95 % CI \|  \|  \| \| Total white matter sk. \| MD \| 0.74 ˑ10^-3^ \| (0.72-0.77) ˑ10^-3^ \| 0.76 ˑ10^-3^ \| (0.74-0.77) ˑ10^-3^ \| 0.37 \| 0.042 \| \| FA \| 0.46 \| (0.44-0.48) \| 0.45 \| (0.44-0.46) \| 0.49 \| 0.025 \| \| Cingulum l.h. \| MD \| 0.73 ˑ10^-3^ \| (0.56-0.90) ˑ10^-3^ \| 0.76 ˑ10^-3^ \| (0.69-0.83) ˑ10^-3^ \| 0.75 \| 0.006 \| \| FA \| 0.76 \| (0.62-0.90) \| 0.70 \| (0.64-0.76) \| 0.46 \| 0.029 \| \| Cingulum r.h. \| MD \| 0.74 ˑ10^-3^ \| (0.60-0.90) ˑ10^-3^ \| 0.76 ˑ10^-3^ \| (0.70-0.82) ˑ10^-3^ \| 0.80 \| 0.004 \| \| FA \| 0.64 \| (0.52-0.75) \| 0.61 \| (0.56-0.66) \| 0.70 \| 0.009 \| \| Unicinate fascicle l.h. \| MD \| 0.92 ˑ10^-3^ \| (0.85-1.0) ˑ10^-3^ \| 0.89 ˑ10^-3^ \| (0.86-0.92) ˑ10^-3^ \| 0.45 \| 0.030 \| \| FA \| 0.35 \| (0.28-0.42) \| 0.37 \| (0.34-0.40) \| 0.55 \| 0.019 \| \| Unicinate fascicle r.h. \| MD \| 0.91 ˑ10^-3^ \| (0.84-0.97) ˑ10^-3^ \| 0.91 ˑ10^-3^ \| (0.88-0.94) ˑ10^-3^ \| 0.88 \| 0.001 \| \| FA \| 0.34 \| (0.28-0.41) \| 0.34 \| (0.32-0.37) \| 0.95 \| 0.000 \| \| **DKI** \|  \| Mean^c^ \| 95 % CI \| Mean^c^ \| 95 % CI \|  \|  \| \| Unicinate fascicle l.h. \| MK \| 0.75 \| (0.68-0.82) \| 0.77 \| (0.74-0.80) \| 0.56 \| 0.018 \| \| Unicinate fascicle r.h. \| MK \| 0.74 \| (0.68-0.80) \| 0.76 \| (0.74-0.79) \| 0.48 \| 0.026 \| \| Hippocampus l.h. \| MK \| 0.67 \| (0.64-0.70) \| 0.67 \| (0.66-0.68) \| 0.92 \| 0.001 \| \| Hippocampus r.h. \| MK \| 0.69 \| (0.66-0.71) \| 0.68 \| (0.67-0.69) \| 0.49 \| 0.026 \|   **Supplementary table 4.** Brain MRI: Volume of brain structures, DTI and DKI measures at follow-up.  Ab; antibody, CI; confidence interval, DKI; diffusion kurtosis imaging, DTI; diffusion tensor imaging, FA; fractional anisotrophy, l.h.; left hemisphere, MD; mean diffusity, MK; mean kurtosis, MRI; magnetic resonance imaging, r.h.; right hemisphere, SEM; standard error of mean, sk; skeleton.  Significance level 0.05. ^a^adjusted for age, sex and estimated intracranial volume. ^b^limbic system (amygdala, parahippocampal cortex and cingulate cortex). ^c^adjusted for age and sex. |
| --- | --- | --- | --- | --- | --- | --- | --- | --- | --- | --- | --- | --- | --- | --- | --- | --- | --- | --- | --- | --- | --- | --- | --- | --- | --- | --- | --- | --- | --- | --- | --- | --- | --- | --- | --- | --- | --- | --- | --- | --- | --- | --- | --- | --- | --- | --- | --- | --- | --- | --- | --- | --- | --- | --- | --- | --- | --- | --- | --- | --- | --- | --- | --- | --- | --- | --- | --- | --- | --- | --- | --- | --- | --- | --- | --- | --- | --- | --- | --- | --- | --- | --- | --- | --- | --- | --- | --- | --- | --- | --- | --- | --- | --- | --- | --- | --- | --- | --- | --- | --- | --- | --- | --- | --- | --- | --- | --- | --- | --- | --- | --- | --- | --- | --- | --- | --- | --- | --- | --- | --- | --- | --- | --- | --- | --- | --- | --- | --- | --- | --- | --- | --- | --- | --- | --- | --- | --- | --- | --- | --- | --- | --- | --- | --- | --- | --- | --- | --- | --- | --- | --- | --- | --- | --- | --- | --- | --- | --- | --- | --- | --- | --- | --- | --- | --- | --- | --- | --- | --- | --- | --- | --- | --- | --- | --- | --- | --- | --- | --- | --- | --- | --- | --- | --- | --- | --- | --- | --- | --- | --- | --- | --- | --- | --- | --- | --- | --- | --- | --- | --- | --- |

| Brain MRI abnormalities | \| Ab-positive  patients (n=7) \| \| --- \| | Ab-negative patients (n=17) |
| --- | --- | --- | --- |
| Cavernous hemangioma | 0 | 1 |
| White matter lesions | 0 | 1 |
| Reduced white matter and thin corpus callosum | 0 | 1 |
| Old infarction | 0 | 1 |
| Normal^a^ | 7 | 13 |

**Supplementary table 5.** Brain MRI abnormalities.

Ab; antibody, MRI; magnetic resonance imaging. ^a^includes smaller white matter lesions regarded as age

appropriate in four antibody-negative patients and a small iron deposit in one antibody-positive and one

antibody-negative patient.

|  | Delta band electrodes | |  | Theta band electrodes | |  | Alpha band electrodes | |  |
| --- | --- | --- | --- | --- | --- | --- | --- | --- | --- |
|  | Ab-positive patients (n=7) | Ab-negative patients (n=19) | | Ab-positive patients (n=7) | Ab-negative patients (n=19) | | Ab-positive patients (n=7) | Ab-negative patients (n=19) | |
|  | Mean (-/+ SD) uV | Mean (-/+ SD) uV | P-value^a^ | Mean (-/+ SD) uV | Mean (-/+ SD) uV | P-value^a^ | Mean (-/+ SD) uV | Mean (-/+ SD) uV | P-value^a^ |
| F3 | 5.29 (3.98,7.02) | 6.11 (4.55,8.21) | 0.29 | 4.09 (3.03,5.52) | 4.73 (3.34,6.71) | 0.36 | 4.62 (2.76,7.72) | 5.48 (3.57,8.40) | 0.42 |
| F4 | 5.91 (4.09,8.53) | 6.21 (4.87,7.92) | 0.70 | 4.28 (3.14,5.83) | 4.83 (3.41,6.84) | 0.44 | 4.85 (3.02,7.80) | 5.56 (3.57,8.67) | 0.52 |
| F7 | 7.54 (5.76,9.88) | 8.57 (5.95,12.35) | 0.42 | 4.78 (3.73,6.13) | 5.13 (3.67,7.16) | 0.63 | 5.26 (3.49,7.94) | 5.74 (3.93,8.37) | 0.63 |
| F8 | 8.08 (6.07,10.76) | 9.16 (6.28,13.35) | 0.45 | 4.70 (3.62,6.10) | 5.53 (4.07,7.52) | 0.24 | 5.26 (3.72,7.42) | 5.81 (3.98,8.48) | 0.56 |
| T1 | 8.80 (6.35,12.21) | 9.55 (7.61,11.97) | 0.50 | 5.18 (4.02,6.66) | 5.65 (4.38,7.29) | 0.46 | 5.63 (3.74,8.48) | 6.00 (4.26,8.45) | 0.71 |
| T2 | 8.63 (6.20,12.00) | 9.50 (7.18,12.58) | 0.48 | 5.22 (4.09,6.67) | 6.13 (4.51,8.33) | 0.24 | 5.63 (4.04,7.85) | 6.14 (4.37,8.64) | 0.58 |
| T3 | 6.06 (4.52,8.12) | 5.52 (4.17,7.30) | 0.48 | 3.99 (2.98,5.35) | 4.39 (3.18,6.05) | 0.52 | 4.91 (3.08,7.81) | 5.38 (3.73,7.76) | 0.62 |
| T4 | 5.52 (4.38,6.97) | 5.21 (3.99,6.82) | 0.63 | 3.82 (2.97,4.92) | 4.37 (3.05,6.27) | 0.39 | 4.97 (3.37,7.35) | 5.41 (3.68,7.95) | 0.64 |
| T5 | 5.78 (4.03,8.29) | 5.73 (4.23,7.77) | 0.96 | 4.30 (2.90,6.37) | 5.22 (3.30,8.24) | 0.35 | 6.09 (3.72,9.97) | 7.04 (4.11,12.05) | 0.56 |
| T6 | 6.02 (4.31,8.40) | 5.73 (4.12,7.96) | 0.75 | 4.65 (2.95,7.31) | 5.23 (3.24,8.44) | 0.59 | 6.53 (3.95,10.81) | 7.75 (4.42,13.59) | 0.50 |
| C3 | 4.79 (3.75,6.11) | 4.76 (3.72,6.10) | 0.96 | 3.81 (2.83,5.14) | 4.31 (3.06,6.06) | 0.43 | 4.19 (2.53,6.92) | 4.94 (3.16,7.75) | 0.44 |
| C4 | 4.83 (3.65,6.38) | 4.61 (3.61,5.89) | 0.70 | 4.04 (2.94,5.56) | 4.19 (2.91,6.03) | 0.83 | 4.22 (2.59,6.88) | 4.95 (3.12,7.85) | 0.47 |
| P3 | 5.41 (4.19,6.99) | 5.45 (4.45,6.67) | 0.95 | 4.10 (2.83,5.94) | 4.63 (3.25,6.59) | 0.47 | 5.13 (3.10,8.48) | 6.10 (3.59,10.38) | 0.48 |
| P4 | 5.96 (4.72,7.54) | 5.57 (4.33,7.16) | 0.55 | 4.35 (3.05,6.20) | 4.52 (3.08,6.62) | 0.83 | 5.40 (3.11,9.38) | 6.33 (3.72,10.76) | 0.53 |
| O1 | 7.14 (5.25,9.73) | 7.28 (5.39,9.83) | 0.90 | 5.64 (3.59,8.86) | 6.37 (3.97,10.22) | 0.58 | 9.52 (5.52,16.41) | 10.49 (5.87,18.75) | 0.71 |
| O2 | 7.44 (5.60,9.87) | 7.01 (5.19,9.47) | 0.67 | 5.98 (3.60,9.94) | 6.09 (3.94,9.43) | 0.93 | 9.42 (5.11,17.34) | 10.74 (6.00,19.20) | 0.63 |
| Fz | 6.12 (4.74,7.90) | 6.12 (4.69,7.98) | 1.00 | 4.81 (3.67,6.31) | 5.33 (3.69,7.70) | 0.53 | 5.01 (3.04,8.27) | 5.85 (3.69,9.26) | 0.49 |
| Cz | 5.72 (4.66,7.03) | 6.04 (4.63,7.89) | 0.64 | 5.12 (4.12,6.35) | 5.62 (4.00,7.91) | 0.52 | 4.85 (3.10,7.57) | 5.79 (3.79,8.84) | 0.38 |
| Pz | 6.57 (5.12,8.45) | 6.41 (5.24,7.85) | 0.81 | 4.72 (3.46,6.42) | 5.11 (3.66,7.14) | 0.60 | 6.02 (3.64,9.94) | 6.43 (3.95,10.47) | 0.77 |

**Supplementary table 6.** qEEG band amplitudes for each electrode separately

^a^Two-sample Student's t-test (LN-transformed amplitudes, retransformed to uV scale)

| **MRI sequence** | **Direction** | **Mode** | **Voxel size** (mm) | **No of slices** | **NSA** | **TR**  (ms) | **TE** (ms) | **TI** (ms) | **Flip-angle** | **Gap**  (mm) | **No of directions** | | **B0**  (s/mm^2^) | | **B1**  (s/mm^2^) | | **B2**  (s/mm^2^) | |  |
| --- | --- | --- | --- | --- | --- | --- | --- | --- | --- | --- | --- | --- | --- | --- | --- | --- | --- | --- | --- |
| **MPRAGE^a^** | Sagittal | 3D | 1.0x1.0x1.0 | 176 | 1 | 230 | 2.01 | 900 | 9° | 0 | | - | | - | | - | | - | |
| **FLAIR** | Sagittal | 3D | 1.0x1.0x1.0 | 192 | 1 | 5000 | 388.00 | 1800 | T2 var | 0 | | - | | - | | - | | - | |
| **SWI** | Axial | 3D | 1.0x0.5x2.0 | 72 | 1 | 29 | 20.0 | - | - | 0 | | - | | - | | - | | - | |
| **T2W** | Axial | 2D | 0.7x0.5x3.0 | 42 | 2 | 4200 | 81.00 | - | 150° | 0 | | - | | - | | - | | - | |
| **DWI** | Axial | 2D | 1.8x1.8x4.0 | 31 | 3 | 6400 | 76.00 | - | - | 0 | | 3 | | 0 | | 1000 | | - | |
| **DTI B0 AP/PA** | Axial | 2D | 3.0x3.0x3.0 | 45 | 1 | 6000 | 90.00 | - | - | 0 | | 20 | | 0 | | - | | - | |
| **DTI** | Axial | 2D | 3.0x3.0x3.0 | 45 | 3 | 6000 | 90.00 | - | - | 0 | | 20 | | 0 | | 1000 | | - | |
| **DKI** | Axial | 2D | 3.0x3.0x3.0 | 20 | 2 | 6000 | 93.00 | - | - | 0 | | 20 | | 0 | | 1000 | | 2000 | |

**Supplementary table 7.** MRI scan protocol below with scan parameters. MPRAGE; magnetization-prepared rapid gradient-echo, FLAIR; fluid attenuated inversion recovery, SWI; susceptibility weighted imaging, T2W; T2 weighted, DWI; diffusion weighted imaging, DTI; diffusion tensor imaging, DTI B0 AP/PA correction scans implementing AP, anterior-posterior phase encoding direction (1 scan); PA, posterior-anterior phase encoding direction (1 scan) according to Holland et al.^1^, DKI; diffusion kurtosis imaging, No; number, NSA; number of signal averages, TR; repetition time, TE; echo time, TI; inversion time.^a^based on the Alzheimer’s disease neuroimaging initiative (ADNI) protocol (see http://adni.loni.usc.edu/methods/documents/mri-protocols/)

Supplementary methods of MRI data acquisition

The cingulum ROI was made by manually drawing 1x4 voxels (12 mm^3^) on one axial slice on each individual FA map where cingulum appeared longest (Figure 2B). Mean FA, MD, RD and AD were extracted from the ROI for each individual. The uncinate fascicle ROI was defined by the ICBM-DTI-81white-matter labels atlas^2^ in MNI space. Registrations were performed between each individual FA map and the FMRIB_FA template given in FSL with the use of FLIRT (linear registrations) and FNIRT (non-linear registrations) in FSL. The inverted transform was used to convert the ROIs into each individual FA-space, and finally mean DTI and DKI parameters were extracted from the ROI. The hippocampus ROI covered the entire hippocampal formation and was drawn manually on the T1-weighted MRI of each individual (Figure 2A). Registrations were performed with FLIRT and FNIRT to register the FA-map to the T1-weighted MRI for each individual. The hippocampus ROIs were transformed from T1-space to FA-space using the inverted FA-to-T1 transform, and were finally used to extract the mean DTI and DKI parameters from the ROI.

References

1 Holland, D., Kuperman, J. M. & Dale, A. M. Efficient correction of inhomogeneous static magnetic field-induced distortion in Echo Planar Imaging. *NeuroImage* **50**, 175-183, doi:10.1016/j.neuroimage.2009.11.044 (2010).

2 Mori, S. *et al.* Stereotaxic white matter atlas based on diffusion tensor imaging in an ICBM template. *NeuroImage* **40**, 570-582, doi:10.1016/j.neuroimage.2007.12.035 (2008).
